# Degradation of Graphene in High- and Low-Humidity Air, and Vacuum Conditions at 300–500 K

**DOI:** 10.3390/nano14020166

**Published:** 2024-01-12

**Authors:** Shunsuke Kawabata, Ryuichi Seki, Takumi Watanabe, Tomonori Ohba

**Affiliations:** Graduate School of Science, Chiba University, 1-33 Yayoi, Inage, Chiba 263-8522, Japan

**Keywords:** graphene, natural degradation, defects, oxidation, thermal stability

## Abstract

Graphene is a fundamental unit of carbon materials and, thus, primary sp^2^-bonded carbon material. Graphene is, however, easily broken macroscopically despite high mechanical strength, although its natural degradation has rarely been considered. In this work, we evaluate the natural degradation of two-layer graphene in vacuo, in low-humidity air, and in high-humidity air at 300, 400, 450, and 500 K. Over 1000 days of degradation at 300 K, the graphene structure was highly maintained in vacuo, whereas the layer number of graphene tended to decrease in high- and low-humidity air. Water was slightly reacted/chemisorbed on graphene to form surface oxygen groups at 300 K. At 450 and 500 K, graphene was moderately volatilized in vacuo and was obviously oxidized in high- and low-humidity air. Surprisingly, the oxidation of graphene was more suppressed in the high-humidity air than in the low-humidity air, indicating that water worked as an anti-oxidizer of graphene by preventing the chemisorption of oxygen on the graphene surface.

## 1. Introduction

Graphene is a two-dimensional planar molecule composed of sp^2^ carbon atoms that was first produced via the exfoliation of graphite by Geim [1]. Graphene offers many extraordinary properties, such as high electrical conductivity, high thermal conductivity, optical transmittance, and flexibility, which have been exploited in various fields, including electronics, electrical engineering, energy devices, and biotechnologies [2,3,4,5,6,7,8,9,10]. Graphene-based nanomaterials are also applied in gas- and bio-sensors [11,12,13,14,15,16,17,18,19]. The exfoliation of graphite to graphene is less mass production technique and generates a considerable number of defects. Graphene obtained via graphene oxide reduction, which is a mass-production technology, contains many defects and large surface roughness [20], whereas the chemical vapor deposition of carbon sources produces highly crystalline graphene. SiC single-crystal surfaces yield highly crystalline multi-layer graphene suitable for semiconductor usage, but the synthesis process is cost-consuming [21,22]. On the other hand, the chemical vapor deposition of carbon sources such as methane, benzene, ethane, acetylene, or toluene on a copper, nickel, or SiO_2_ substrate has the advantage of uniform large-area graphene, which is well used as an efficient graphene-production technique [1,23,24,25,26,27,28,29,30,31]. Graphene on a hydrophilic SiO_2_ substrate with OH groups is, however, highly reactive and degrades under ultraviolet (UV) light, but graphene on a bare SiO_2_ substrate is unchanged [32]. Graphene defects are also created via electron beam irradiation at energies below 80 eV [33], and graphene nanoribbons are damaged by Joule heating despite having high mechanical strength [3]. However, to the best of our knowledge, the natural degradation of graphene has not been investigated, because it requires a long-term investigation. Here, we first demonstrate the natural degradation of graphene in various environments (in low- and high-humidity air and in vacuo at 300, 400, 450, and 500 K) and under visible laser irradiation for a long period.

## 2. Experimental and Simulation Procedure

### 2.1. Materials

Two-layer graphene was synthesized via chemical vapor deposition of CH_4_ on Cu foil (80 μm thickness, CU-113303, Nilaco Co., Tokyo, Japan). During the first synthesis stage, the Cu substrate was placed in a quartz tube in a furnace, then heated to 1300 K at 33 K min^−1^. For annealing, the Cu substrate was maintained at that temperature for 30 min in a reductive gas (H_2_) and a carrier gas (Ar) with gas flow rates of 20 and 500 cm^3^ min^−1^, respectively. Graphene was then synthesized at 1300 K for 30 min in CH_4_ (0.2 cm^3^ min^−1^), H_2_ (20 cm^3^ min^−1^), and Ar (500 cm^3^ min^−1^). The synthesized graphene was cooled down to below 400 K in H_2_ (20 cm^3^ min^−1^) and Ar (500 cm^3^ min^−1^) gas flows. Polycarbonate (46 mg mL^−1^) in CHCl_3_ solution was spin-coated on the graphene synthesized on the Cu substrate, and the graphene samples were then heated at 400 K for 10 min in air. The underlying Cu substrate was dissolved in 50 mL of a 0.1 mol dm^−3^ (NH_4_) S_2_O_8_ solution, and the graphene samples were carefully placed on distilled water for 1 h to remove those ions. The samples were finally transferred onto platinum and quartz.

### 2.2. Natural Degradation Conditions

The synthesized graphene samples were exposed to low-humidity air (10–30% relative humidity), high-humidity air (80% relative humidity), and vacuum conditions (below 1 kPa) at 300 ± 5 K. The 80% relative humidity condition was controlled with saturated KBr aqueous solution at 300 ± 5 K [34]. The graphene-exposure experiments were also conducted at 400 ± 5, 450 ± 5, and 500 ± 5 K. Water vapor was introduced into the stainless-steel cell until reaching 3.1 kPa and 300 K, corresponding to 80% relative humidity, and graphene was then heated at 400, 450, and 500 K for 40, 25, and 13 days, respectively. The other exposure conditions were the same as those mentioned above.

### 2.3. Characterizations

The layer numbers and crystallinity changes during the natural degradation of graphene were evaluated from Raman scattering spectra of the graphene samples (laser irradiation = 532 nm, 0.3 mW for 1 min) (NRS-4500, JASCO Co., Tokyo, Japan). Seven data points were collected for each Raman scattering measurement. To check the graphene stability under laser irradiation, the samples were irradiated with the 532 nm 0.3 mW laser for 0 to 300 min prior to natural degradation tests. The visible reflectance spectra of graphene were obtained using UV–visible light micro-spectroscopy (MSV-370, JASCO Co., Tokyo, Japan). The structural changes in the exposed graphene were evaluated via X-ray photoelectron spectroscopy (XPS) (JPS-9030, JEOL Co., Tokyo, Japan) at 10 kV and 10 mA using Mg Kα radiation at <10^−6^ Pa. The XPS spectra were deconvoluted with a Gaussian–Lorentzian fitting function.

### 2.4. MD Simulations

Thermal fluctuation and degradation of 30 nm graphene was evaluated from the molecular dynamics (MD) simulation using the leapfrog Verlet algorithm, which is a laboratory-made program. The intramolecular and intermolecular interactions were calculated using the environment-dependent interatomic and Lennard-Jones potentials with σ = 0.34 nm and ε = 28.0 K, respectively [35,36,37]. Single-layer graphene of 30 nm in diameter, named as nano-graphene, was set parallel on *z* = 0.4 nm, and carbon atoms were moved according to the above interaction potentials. Nano-graphene was also interacted with fixed imaginary graphene with Lennard-Jones potential well depth of 2.8 K on *z* = 0.0 nm, which was used as a substrate. The unit cell size was 50 × 50 × 5 nm^3^. The time step and accumulation time were 0.1 fs and 10 ps. The temperature of solid carbon atoms was controlled to less than 0.1 K during the first stabilization for 10 ps and then maintained at 300, 400, 450, or 500 K using the heat bath coupling method.

## 3. Results and Discussion

### 3.1. Graphene Degradation at 300 K

**Figure 1:**
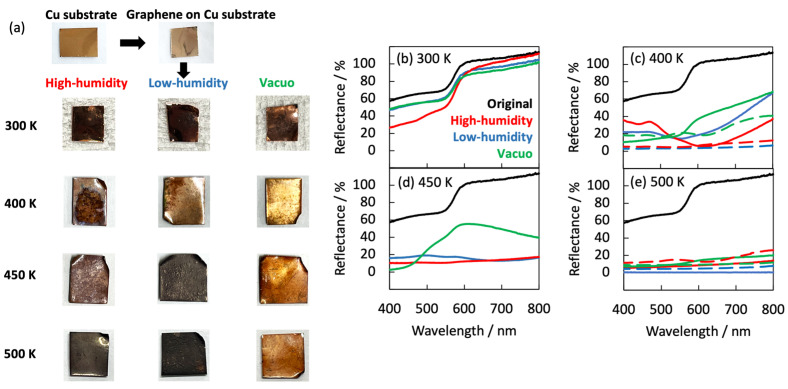
(**a**) Optical images of the graphene samples in high- and low-humidity air and in vacuo at 300, 400, 450, and 500 K. Optical images of copper at 500 K are shown for comparison. Visible reflectance spectra of graphene after 420 days at 300 K (**b**), 40 days at 400 K (**c**), 25 days at 450 K (**d**), and 13 days at 500 K (**e**) in high-humidity air (red), in low-humidity air (blue), in vacuo (green), and a bare copper substrate (dotted curves). The visible reflectance spectra of as-prepared graphene (black) are shown for comparison.

**Figure 2:**
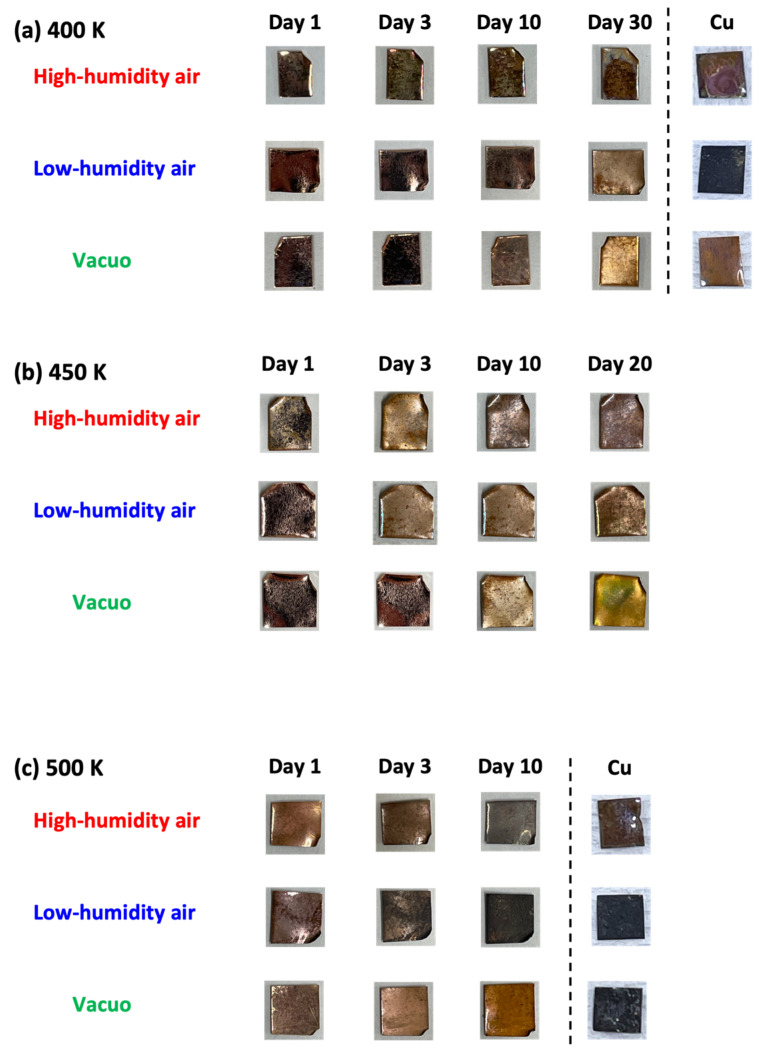
Optical images of graphene samples in high-humidity air, low-humidity air and vacuo at 400 (**a**), 450 (**b**), and 500 K (**c**).

The natural degradation of graphene was evaluated from the optimal images and visible reflectance spectra (see Figure 1 and Figure 2). The graphene samples on the Cu substrate appeared shiny after 420 days in vacuo at 300 K, but matte after exposure to high- and low-humidity air. At higher temperatures (400, 450, and 500 K), the graphene samples on the Cu substrate became lusterless under both air conditions. The samples in low-humidity air at 400, 450, and 500 K were darkened via the oxidation of copper. Surprisingly, the graphene samples exposed to 400 and 500 K for 10 days became glossy in high-humidity air and matte in low-humidity air. Between 10 and 40 days at 400 K, the graphene surface became rough and at 450, and 500 K, the copper substrates were obviously oxidized in both low- and high-humidity air. The samples were partially oxidized in low-humidity air at 400, 450, and 500 K, although the copper surface coated with graphene was apparently less oxidized. The darkened sample color was attributed to the partial disappearance of graphene in low-humidity air, as described later. In contrast, the samples maintained in high-humidity air maintained their shiny appearance (Figure 1a and Figure 2). These visual features were mirrored in the reflection spectra of the samples (Figure 1b–e). The strong reflectance of the as-prepared graphene sample at wavelengths above 550 nm was attributed to copper reflection. The reflectance was almost maintained at 300 K, but was considerably reduced in the wavelength <550 nm region in high-humidity air (Figure 1b). The high reflectance was kept when graphene covered the copper substrate well and prevented the copper from being oxidized. Therefore, graphene was maintained in low-humidity air and vacuo, while graphene was partially oxidized in high-humidity air at 300 K. Above 400 K, the reflectance in high-humidity and low-humidity air and in vacuo was decreased in all wavelength regions. However, the reflectance at 400 K was obviously higher than the bare copper substrates, proposing that graphene mostly covered copper substrates (Figure 1c). The maintenance rates of graphene were in the order of in vacuo, in low-humidity air, and in high-humidity air conditions at 400 K. The trend at 450 K was similar to that at 400 K; the reflectance in vacuo, at 450 K, was still high, while those in high-humidity and low-humidity were considerably low, as expected from the optical images (Figure 1d). The reflectance at 500 K was very low in all the conditions owing to graphene removal from the copper substrate, because the reflectance was similar to that of the bare copper substrate (Figure 1e). The anomalies in the optical images and the reflectance spectra of graphene clearly depended on both humidity and temperature.

**Figure 3:**
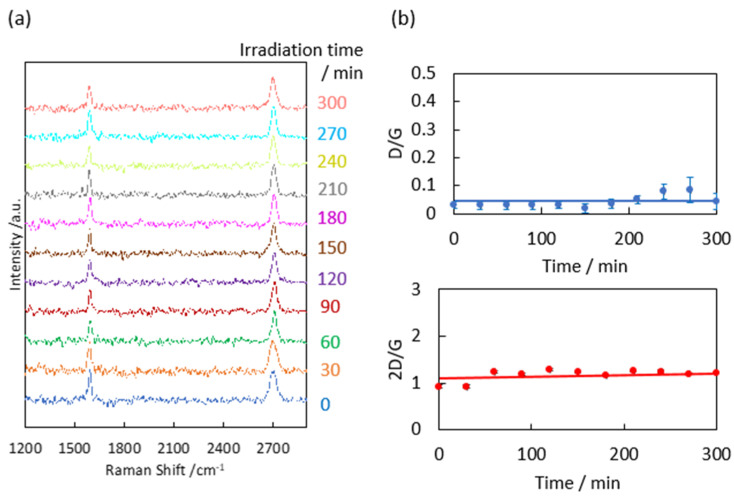
(**a**) Raman scattering spectra of graphene after laser irradiation at 0.3 mW. (**b**) D/G and 2D/G band ratios as a function of the irradiation time.

To clarify the link between the optical features and structures of the graphene samples, the Raman scattering spectra of the samples constitute the standard method of observing graphene structures from D, G, and 2D bands at 1350, 1580, and 2680 cm^−1^, respectively. We first confirmed that graphene was hardly damaged by laser irradiation during the Raman scattering measurements prior to the evaluation of natural graphene degradation. The graphene degradation by 0.3 mW visible laser irradiation for 0–300 min was examined, as shown in Figure 3. We here assume that the D/G and 2D/G band intensities are conventionally related to defects in the graphene and graphene layer number, respectively [38]. The 2D/G band ratio was 0.9 ± 0.03, implying that the as-prepared graphene was two-layer graphene. Meanwhile, the D/G band ratio was 0.03 ± 0.01, indicating that the graphene was highly crystalline [38,39]. The D/G and 2D/G ratios in the Raman scattering measurements were independent of the laser irradiation time (0–300 min; see Figure 3b). To observe natural graphene degradation via Raman scattering, the Raman observations required seven minutes of laser irradiation per step (one minute × seven points on each graphene sample) multiplied by 18 times (up to 1000 days in graphene degradation tests). The accumulated laser irradiation time for each sample became 112 min. As the graphene was undamaged after 300 min of laser irradiation (Figure 3), it was concluded that graphene was hardly damaged by laser irradiation during the Raman scattering measurements.

**Figure 4:**
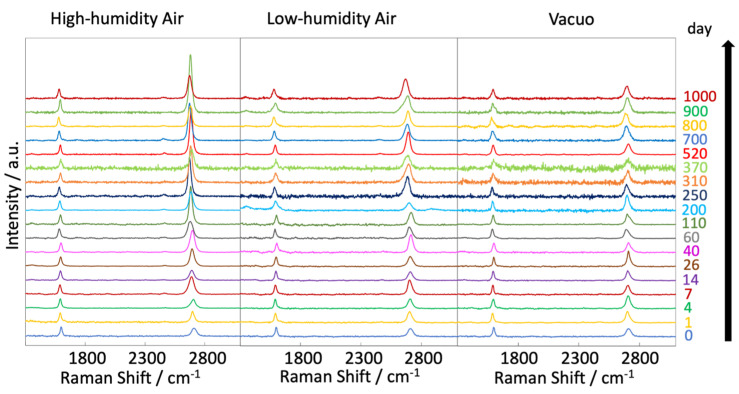
Raman spectra of graphene at 300 K, acquired at different times (0–1000 days) after graphene synthesis in high-humidity air, low-humidity air and vacuo.

**Figure 5:**
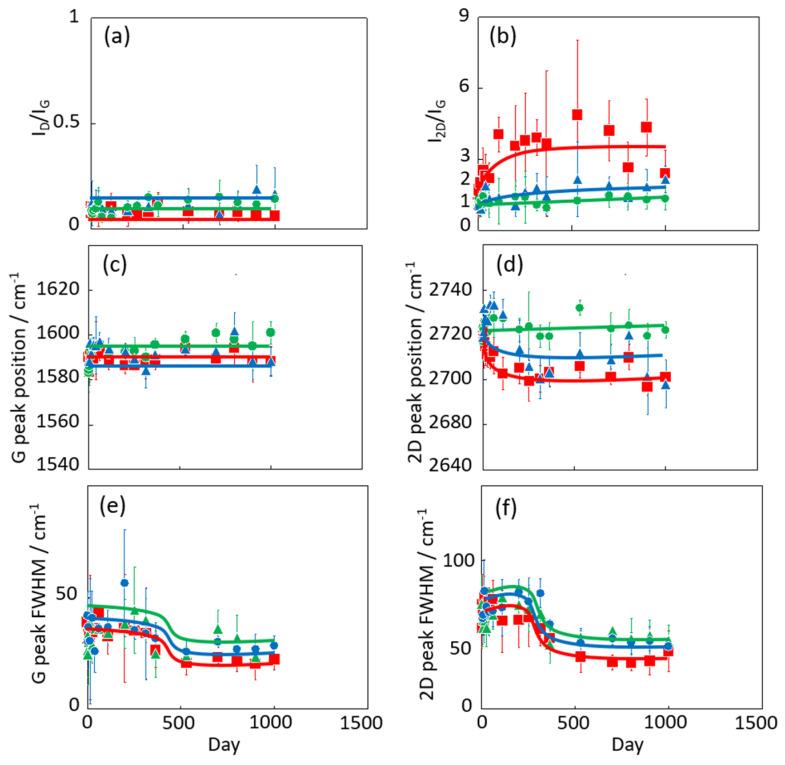
Time dependence of D/G (**a**), 2D/G ratios (**b**), G peak position (**c**), 2D peak position (**d**), FWHM of G bands (**e**), and 2D bands (**f**) in the Raman spectra of graphene in high-humidity air (red), low-humidity air (blue) and vacuo (green). All graphene samples were maintained at 300 K.

Figure 4 shows the Raman scattering spectra of graphene after different exposure times (0–1000 days) at 300 K in vacuo and in low- and high-humidity air. All intensities in each spectrum were normalized by the height of the G band. The G and 2D bands of the samples held in vacuo remained constant over time, and D bands were hardly observed. According to the Raman spectra, the graphene structure was maintained under vacuum conditions. D bands were also hardly observed in the spectra of graphene in air, but 2D bands emerged over time, especially under the high-humidity air condition. Those are clearly shown in Figure 5. This spectral change in 2D bands probably indicates a structural change in graphene through a moderate reaction with water vapor and/or oxygen gas in the air. Oxidation likely influences the graphene nano-structures even at 300 K. Density functional theory calculations indicated an oxidation role of water on graphene via the chemisorption and formation of hydroxyl groups from 473 K [40]. In the present study, the increasing intensity of the 2D bands implies a change from two-layer graphene to single-layer graphene as the upper graphene layer reacted with water vapor at 300 K (as mentioned above). Therefore, the maintenance of the graphene nanostructure requires a low-humidity and/or vacuum environment. The temporal changes in the D/G and 2D/G band ratios are clarified in Figure 5a,b. The D/G band ratios were mostly unchanged over time, ranging from 0.11 (zero days) to 0.14, 0.17, and 0.06 (at 1000 days) in vacuo, in low-humidity air, and in high-humidity air, respectively (Figure 5a). Although the differences in the D/G band ratios were of the nominal level, active sites on the graphene were reacted with water vapor and oxygen gas in the very early stage of the graphene degradation tests. In contrast, the 2D/G ratios gradually increased from 1.1 at zero days to 1.3, 2.2, and 3 at 1000 days in vacuo, in low-humidity air, and in high-humidity air, respectively (Figure 5b). During exposure to high-humidity air, the 2D/G band ratios were increased over time, especially in the first 100 days, while those in low-humidity air and in vacuo were slightly increased. Graphene splitting into its fragments was also observed through reaction with water, while no graphene splitting was observed in oxygen, hydrogen, and ammonia environments [41]. The G peak positions shown in Figure 5c were unchanged in all the conditions. On the other hand, the 2D peak positions in low- and high-humidity air were shifted to the smaller wavenumbers as time progressed, whereas that in vacuo was unchanged (Figure 5d) [42]. These results propose that the graphene structure changes in low- and -high-humidity air, probably with a decrease in graphene layer number. The full width at half maximum (FWHM) of the G and 2D bands indicated the obvious step at 300–500 days (Figure 5e,f). As strain on graphene flakes caused the splitting and broadening of 2D bands, the relaxation of strain on graphene flakes might occur via the partial release of the connection between graphene and the copper substrate [43].

### 3.2. Graphene Degradation at 400, 450, and 500 K

**Figure 6:**
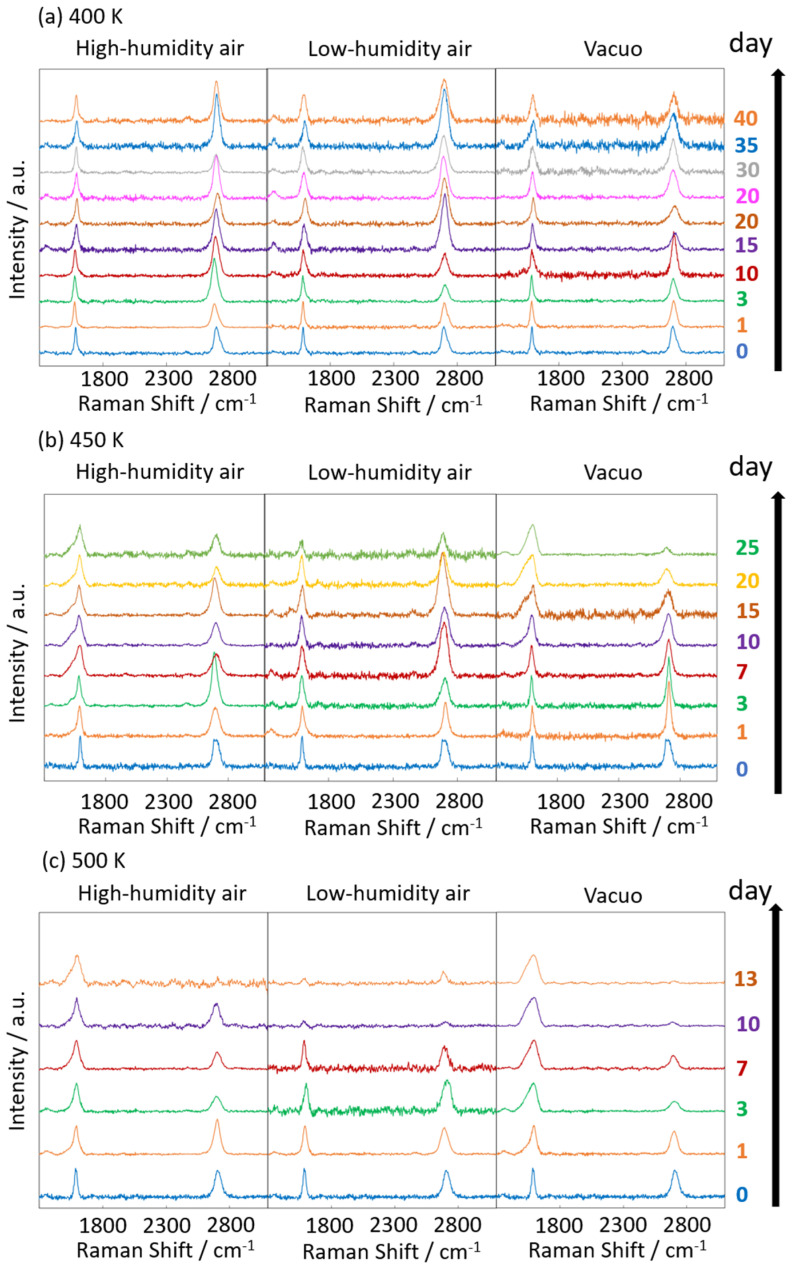
Raman spectra of graphene at 400 (**a**), 450 (**b**), and 500 K (**c**). Spectra were acquired at 0–40 days (400 K), 0–25 days (450 K), and 0–10 days (500 K) after graphene synthesis in low-humidity air, high-humidity air and vacuo.

**Figure 7:**
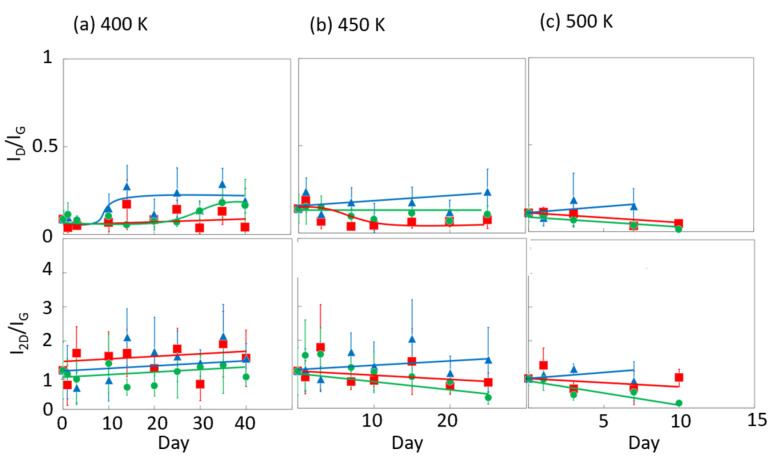
The 2D/G and D/G ratios in the Raman spectra of graphene in high-humidity air (red), low-humidity air (blue) and vacuo (green) at 400 (**a**), 450 (**b**), and 500 K (**c**).

**Figure 8:**
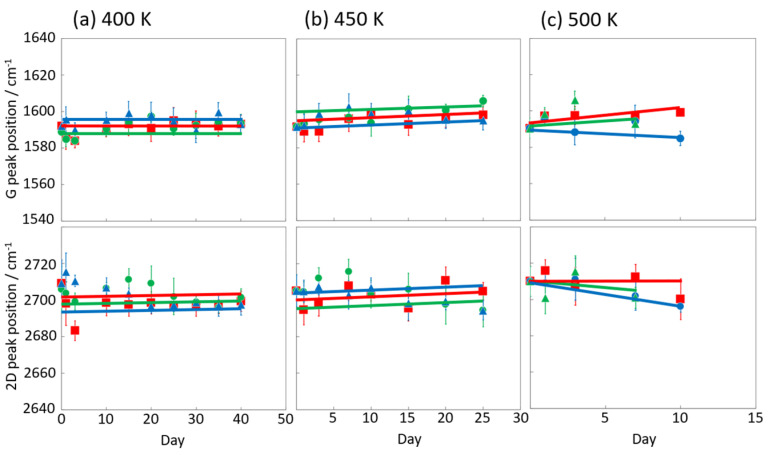
G and 2D peak position in the Raman spectra of graphene in high-humidity air (red), low-humidity air (blue) and vacuo (green) at 400 (**a**), 450 (**b**), and 500 K (**c**).

**Figure 9:**
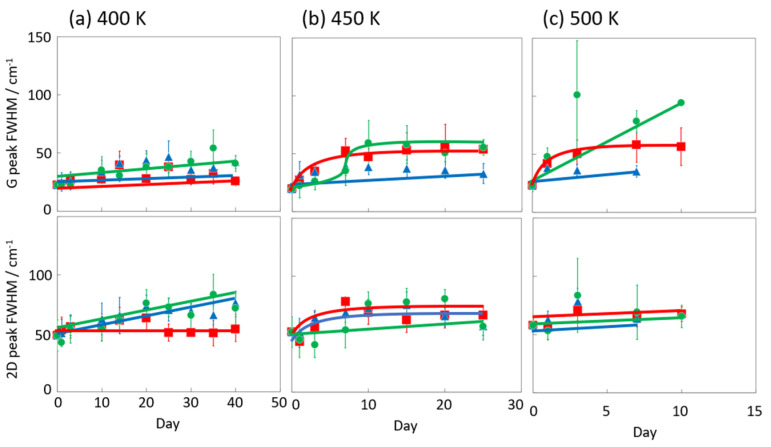
G and 2D peak FWHM in the Raman spectra of graphene in high-humidity air (red), low-humidity air (blue) and vacuo (green) at 400 (**a**), 450 (**b**), and 500 K (**c**).

Figure 6 and Figure 7 display the Raman spectra of graphene during natural degradation, and the D/G and 2D/G peak ratios, respectively, of graphene in vacuo, in low-humidity air, and in high-humidity air at 400, 450 and 500 K. The results on different days exhibited the obvious natural degradation of graphene. The 2D bands were weakened on several days, especially in vacuo. The average D/G band ratios at 400 K ranged from 0.09 at zero days to 0.16, 0.19, and 0.04 at 40 days in vacuo, in low-humidity air, and in high-humidity air, respectively (Figure 6a and Figure 7a). The average D/G band ratios at 450 K were maintained at 0.14 ± 0.1 in vacuo and in high-humidity air, while those in low-humidity air changed from 0.13 ± 0.1 to 0.24 ± 0.1 (Figure 6b and Figure 7b). The D/G band ratios followed the same trend at 500 K: from 0.11 at zero days to 0.01 at 10 days in vacuo, 0.15 at 7 days in low-humidity air, and 0.05 at 10 days in high-humidity air (Figure 6c and Figure 7c). Here, the D/G and 2D/G band ratios at 500 K in 10 and 13 days were not plotted, because the D, G, and 2D bands were hardly observed, but those approximate values were 0.01 at 13 days in vacuo, 0.07 at 13 days in high-humidity air, and 0.31 at 10 days and 0.71 at 13 days in low-humidity air. Under vacuum and high-humidity conditions at 450 K, the G band widened, and the 2D band diminished in 25 days. The spreading of the G band peaks accompanied the appearance of the D’’ peak around 1500 cm^−1^ derived from the low-crystallinity phase [44]. Graphene might gradually volatilize at 500 K in 10 days, while graphene at 400 K was maintained until 40 days in all the conditions. At 450 K, the Raman spectra of graphene was between 400 and 500 K; that is, graphene was partially volatized in 25 days. The 2D/G ratios at 400 K were as follows: 1.1 at zero days to 0.9, 1.5, and 1.5 at 40 days in vacuo, in low-humidity air, and in high-humidity air, respectively (Figure 6a). In contrast, the 2D/G ratios at 450 K were differently changed, from 1.1 at zero days to 0.3, 1.4, and 0.8 at 40 days in vacuo, in low-humidity air, and in high-humidity air, respectively (Figure 6b). The 2D/G ratios at 500 K indicated changes from 0.9 at zero days to 0.14 at 10 days in vacuo, 0.9 at 10 days in high-humidity air, and 0.8 at 7 days in low-humidity air, respectively (Figure 6c). The slight change in the 2D/G band ratio in high-humidity air below 450 K corresponds to the optical image, indicating the maintenance of the graphene structure. The reaction mechanisms associated with graphene degradation at 450 and 500 K might differ from those at 300–400 K. The slight changes in the D/G band ratios at 300 and 400 K in high-humidity air were probably caused by the reaction of graphene with water vapor on the graphene edges, manifested as a slight increase in the D band [45]. The 2D/G band ratios were increased in the order of those in high-humidity air, in low-humidity air, and in vacuo at 300 and 400 K. The systematic changes were caused by water chemisorption reactions that reduced the layer number of graphene. On the other hand, at 450 and 500 K, graphene was volatilized in vacuo, while oxygen gas was reacted/chemisorbed on the graphene under non-vacuum conditions, creating defects (increased intensity of the D band), and graphene was destroyed via a continuous oxidation reaction with CO_2_ and CO production for long-time heating in high- and low-humidity conditions at 500 K. Although defect creation on graphene could be caused by oxidation gas in the low- and high-humidity air, more change in low-humidity air indicated that the oxidation reaction was inhibited by water vapor. This was caused by the quick oxidation of graphene with water vapor [46]. Water chemisorption and oxidation mainly occurred on graphene edges and, thus, competed with each other, and an oxidation reaction via oxygen gas, in which water vapor chemisorbs on graphene, was prevented. The G and 2D peak positions in Figure 8 were hardly changed at 400 and 450 K, whereas those at 400 K in low-humidity air were changed to smaller wavenumbers, indicating a depression in the graphene layer number. Figure 9 shows the FWHM of the G and 2D bands. The FWHM was unexpectedly increased in vacuo and in high-humidity air; this phenomenon was caused by the strain of graphene according to the thermal expansion difference between graphene and the copper substrate. On the other hand, small graphene flakes formed through the oxidation reaction in low-humidity air relaxed the graphene strains [47].

**Figure 10:**
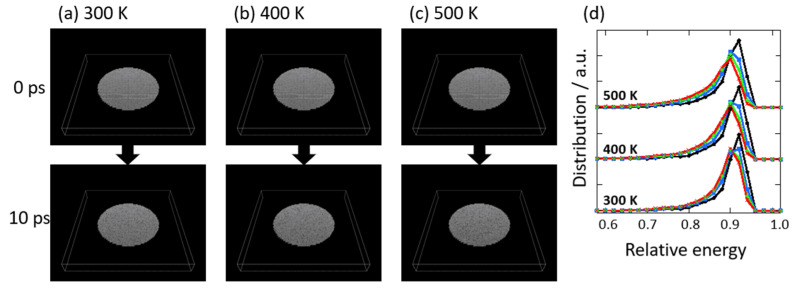
Snapshots of nano-graphene as a function of time progress at 300 (**a**), 400 (**b**), and 500 K (**c**). (**d**) The relative stability distributions of carbon atoms in nano-graphene against perfect graphene at 2.5 (black circle), 5.0 (blue square), 7.5 (green diamond), and 10 ps (red cross).

Graphene degradation in vacuo at 300, 400, and 500 K was evaluated using the MD simulation of nano-graphene. As the nano-graphene of 30 nm in diameter was somewhat smaller than actual graphene, the stability of nano-graphene was expected to be much smaller than the actual graphene. The temperature was assumed to be controlled only by the thermal fluctuations in carbon atoms. Despite the small graphene size, overestimated thermal fluctuations, and short period calculations, the trend of graphene degradation was expected. Figure 10a–c show the snapshots of nano-graphene degraded at 300, 400, and 500 K in vacuo. The nano-graphene had a relatively large amount of edge sites, and graphene structures were more easily deconstructed from these edge carbons, although the snapshots indicated the maintenance of graphene structures. However, the stability of graphene structure was obviously different due to the temperature conditions, as shown in Figure 10d. The averaged relative stability was changed from 0.97 to 0.88 in 10 ps at 300 K, while this were changed to 0.86 at 400 K, and 0.85 at 500 K. At a higher temperature, the distributions with less stability between 0.4 and 0.8 were more increased, because of increasingly less stabilized sites on the edges. Bulat and co-workers also proposed that there is higher reactivity at the edge sites than the basal plane [48]. When a hydrogen atom was added on graphene at once, the adjoined sites were activated. The reaction was continuously carried out. The same feature was observed in our MD simulation. The less-stabilized sites via thermal stimulation propagated the whole structure of graphene. Therefore, the less-stabilized structure at edge sites was first observed in the graphene-degradation process, then the less-stabilized structure was gradually propagated toward the graphene center, and the whole graphene structure was changed to a defective structure with less stability, as expected experimentally.

**Figure 11:**
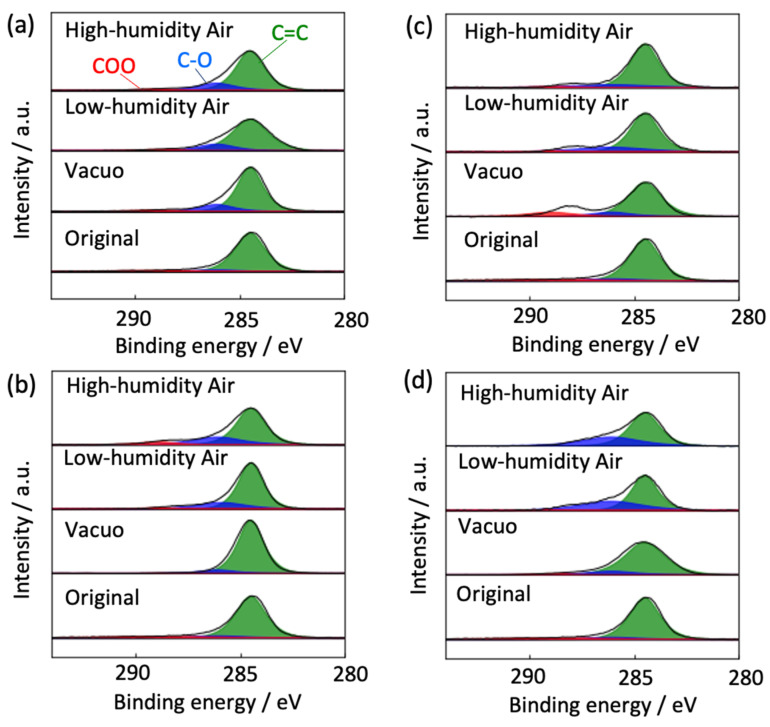
C1s peaks in the XPS of degraded graphene after 370 days at 300 K (**a**), 40 days at 400 K (**b**), 25 days at 450 K (**c**), and 13 days at 500 K (**d**) in high-high humidity air, low-humidity air and vacuo.

The degradation mechanism of graphene was inferred from the chemical structure changes in graphene via XPS (see Figure 11). The as-prepared graphene contained a negligible amount of oxygen functional groups (O/C ratio = 0.17). The number of oxygen functional groups was increased in high- and low-humidity air at 300 K (O/C ratios = 0.21 and 0.21, respectively, versus 0.20 in vacuo). Therefore, water vapor was chemisorbed on graphene as hydroxyl and/or carboxyl groups even at 300 K, consistent with the Raman scattering spectra (Figure 4 and Figure 6). The O/C ratios in vacuo, in low-humidity air, and in high-humidity air were 0.09, 0.24, and 0.30, respectively, at 400 K, and 0.24, 0.18, and 0.26, respectively, at 450 K, and 0.14, 0.39, and 0.34, respectively, at 500 K, although the graphene structures in the high- and low-humidity air at 500 K in 13 days were significantly destroyed, as shown in Figure 6. Graphene was more oxidized in a higher-temperature environment, although the O/C ratio remained low in vacuo. Judging from the larger O/C ratios in high-humidity air than in low-humidity air and the maintenance of D/G band ratios in the high-humidity air in comparison with the low-humidity air (Figure 5 and Figure 7), the antioxidative activity of water arose from competitive water chemisorption at edge sites against oxidation via oxygen gas and subsequent graphene degradation. Anti-oxidation was also observed in the XPS of the Cu 2p_3/2_ peaks. In high- and low-humidity air at 400, 450, and 500 K, copper was oxidized to CuO after graphene oxidation exposed the copper surfaces. This phenomenon was especially observed in low-humidity air, further supporting the anti-oxidizing effects of water on graphene. We thus presumed that the bare copper substrate not covered with graphene was first oxidized, and the graphene edges were then oxidized by reacting with oxidized copper, whereas direct oxidation on the basal plane of graphene hardly occurred in all the conditions. The substrate dependence was discussed in Section 3.3.

**Figure 12:**
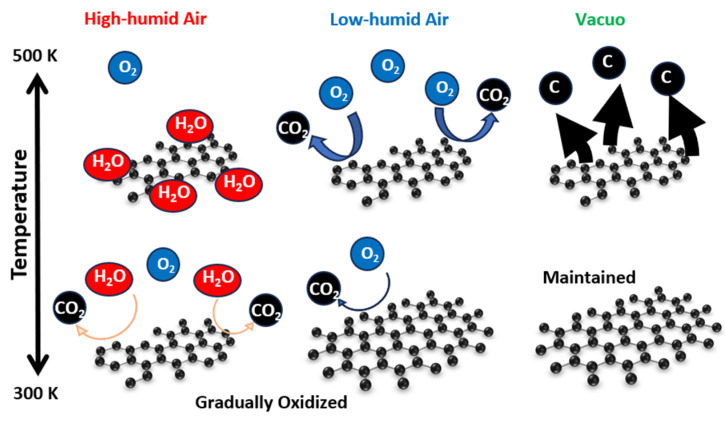
Natural degradation of graphene in high-humidity air, low-humidity air and vacuo.

Figure 12 summarizes the graphene-degradation scheme in high- and low-humidity air and in vacuo at 300–500 K. Graphene structures at the low temperature were maintained in vacuo and hardly changed in low-humidity air and in vacuo, whereas graphene gradually reacted with water vapor in high-humidity air even at 300 K. At the high temperature, graphene was volatilized in vacuo, and oxidized in low-humidity air, while graphene oxidation was prevented in high-humidity air, because chemisorbed water prevented the continuous further oxidation of graphene.

### 3.3. Substrate Effects on Graphene Degradation at 450 K

**Figure 13:**
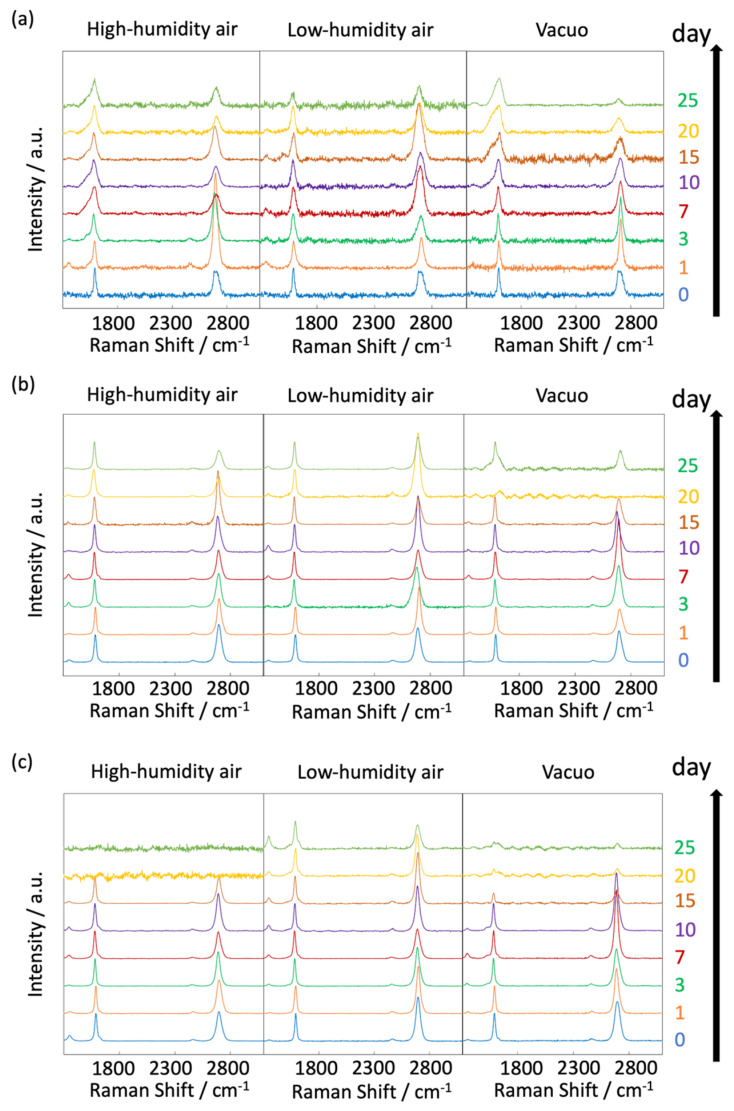
Raman spectra of graphene at 450 on Cu (**a**), Pt (**b**), and quartz (**c**). Spectra were taken after graphene synthesis in low-humidity air, high-humidity air and vacuo.

**Figure 14:**
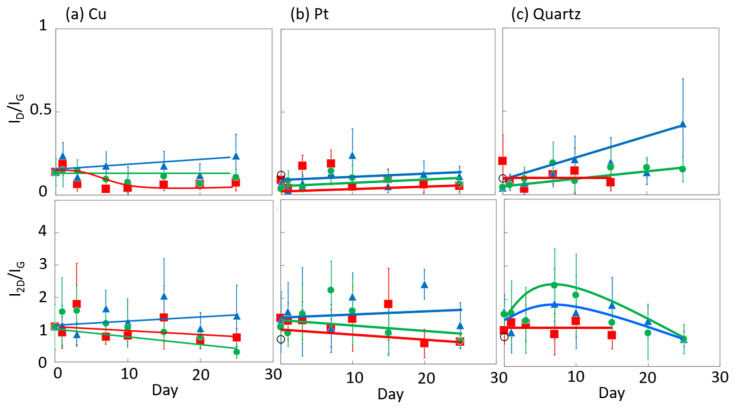
The 2D/G and D/G ratios in the Raman spectra of graphene in high-humidity air (red), low-humidity air (blue) and vacuo (green) at 450 K on Cu (**a**), Pt (**b**), and quartz (**c**).

The influence of graphene degradation caused by different substrates was evaluated using copper, platinum, and quartz substrates. Figure 13 shows the time-dependent Raman scattering of graphene on platinum and quartz substrates at 450 K. The D/G and 2D/G band ratios are summarized in Figure 14. The graphene on the quartz substrate disappeared in the high-humidity air and in vacuo in 20 days, because graphene might be spilled out from the substrate owing to the weak interaction between the graphene and quartz substrate (Figure 13c and Figure 14c). On the other hand, the graphene in the low-humidity air was relatively strongly connected to the quartz substrate, probably via reaction with the surface oxygen on the quartz. We thus evaluated the graphene degradation up to 20 days. The D/G band ratios for both the platinum and quartz substrate hardly changed at 0.1 ± 0.05 in all the conditions at 450 K, except for on the quartz substrate in low-humidity air. Defects on graphene were hardly inserted, although those band ratios in the low-humidity conditions were increased. The oxidation reaction on the quartz substrate was probably promoted by the flexibility of graphene owing to the weak interaction between the graphene and the quartz substrate. Similarly, the 2D/G band ratios agreed with those on copper substrates; the 2D/G band ratios on platinum substrates were changed from 1.1 to 0.7 in vacuo, from 1.4 to 0.7 in high-humidity air, and from 1.3 to 1.2 in low-humidity air, and the 2D/G band ratios on quartz substrates changed from 1.6 to 0.8 via the maximum at 2.4 in vacuo, from 1.1 to 0.9 in high-humidity air, and from 1.6 to 0.8 via the maximum at 1.8 in low-humidity air. The results for the quartz substrate also proposed that the high-humidity air condition prevented graphene degradation by adsorbed water. Copper and Pt substrates rarely influenced graphene stability, although a copper substrate is a relatively reactive material. On the other hand, the graphene on the quartz substrate was more reactive owing to the weak interaction between the graphene and the quartz substrate. In addition, damage during the graphene transfer process was avoidable, because the transferred graphene film maintained its D/G and 2D/G band ratios, high electrical conductivity, and high light transmission [49].

## 4. Conclusions

We demonstrated the natural degradation of graphene in high-humidity air, in low-humidity air, and in vacuo at 300 K up to 1000 days, 400 K up to 40 days, 450 K up to 25 days, and 500 K up to 13 days. The D/G ratios remained almost constant until 1000 days at 300 K in vacuo, whereas the 2D/G ratios gradually increased from 1.1 at day 0 to 3 at 1000 days in high-humidity air and to 2.2 at 1000 days in low-humidity air. Although the changes in the D/G band ratio were slight, the contents of oxygen functional groups were obviously increased. Thus, water vapor and oxygen gas gradually reacted with graphene without disrupting the honeycomb structure of graphene. The graphene layer number (determined from the 2D/G band ratio) decreased as the upper layer of graphene was gradually volatilized, while the defect amounts created via oxidation at 300 K remained constant. Graphene naturally degraded in air at 400, 450, and 500 K, while graphene was stable under vacuum conditions below 450 K. Graphene and its copper substrate were both oxidized in the high- and low-humidity air conditions. However, graphene and copper substrates were more mildly oxidized in the high-humidity air than in the low-humidity air. This indicated that water vapor was preferentially chemisorbed on reaction sites in graphene and prevented any further oxidation reaction on graphene from oxygen gas. Therefore, the chemisorption of water vapor on graphene inhibited the natural degradation of graphene, even in air. To summarize the results obtained in this work: (1) the graphene structure was maintained in vacuo at 300 K, while those in air were partially degraded; (2) graphene was moderately volatilized in vacuo between 400 and 500 K; and (3) water vapor prevented sequential oxidation by oxygen gas at 400–500 K in high-humidity air. To the best of our knowledge, the natural degradation of graphene under various conditions has not been previously clarified. Our findings are expected to contribute significantly to graphene-related sciences. Further studies on the natural degradation of graphene are necessary in various gas environments, especially in pure gas and via molecular simulation, as well as ab initio calculation.

## Data Availability

Data are contained within the article.
